# Mapping brain activity of gut-brain signaling to appetite and satiety in healthy adults: A systematic review and functional neuroimaging meta-analysis

**DOI:** 10.1016/j.neubiorev.2022.104603

**Published:** 2022-05

**Authors:** Sarah Althubeati, Amanda Avery, Christopher R. Tench, Dileep N. Lobo, Andrew Salter, Sally Eldeghaidy

**Affiliations:** a Division of Food, Nutrition & Dietetics, School of Biosciences, University of Nottingham, Loughborough LE12 5RD, UK; b Faculty of Applied Medical Sciences, Department of Clinical Nutrition, King Abdulaziz University, Jeddah, Saudi Arabia; c Division of Clinical Neurosciences, Clinical Neurology, University of Nottingham, Queen's Medical Centre, Nottingham, UK; d NIHR Nottingham Biomedical Research Centre, Queen’s Medical Centre, University of Nottingham, Nottingham, UK; e Nottingham Digestive Diseases Centre, National Institute for Health Research (NIHR) Nottingham Biomedical Research Centre, Nottingham University Hospitals NHS Trust and University of Nottingham, Queen’s Medical Centre, Nottingham NG7 2UH, UK; f MRC Versus Arthritis Centre for Musculoskeletal Ageing Research, School of Life Sciences, University of Nottingham, Queen’s Medical Centre, Nottingham NG7 2UH, UK; g Division of Food, Nutrition & Dietetics and Future Food Beacon, School of Biosciences, University of Nottingham, Loughborough LE12 5RD, UK; h Sir Peter Mansfield Imaging Centre, School of Physics and Astronomy, University of Nottingham, Nottingham NG7 2RD, UK

**Keywords:** Gut-brain axis, Satiety, appetite, Gut peptides, Neuroimaging, Coordinate based meta-analysis, Activation likelihood estimation (ALE), Analysis of Brain Coordinates (ABC), Obesity, Altered eating behavior

## Abstract

Understanding how neurohormonal gut-brain signaling regulates appetite and satiety is vital for the development of therapies for obesity and altered eating behavior. However, reported brain areas associated with appetite or satiety regulators show inconsistency across functional neuroimaging studies. The aim of this study was to systematically assess the convergence of brain regions modulated by appetite and satiety regulators. Twenty-five studies were considered for qualitative synthesis, and 14 independent studies (20-experiments) found eligible for coordinate-based neuroimaging meta-analyses across 212 participants and 123 foci. We employed two different meta-analysis approaches. The results from the systematic review revealed the modulation of insula, amygdala, hippocampus, and orbitofrontal cortex (OFC) with appetite regulators, where satiety regulators were more associated with caudate nucleus, hypothalamus, thalamus, putamen, anterior cingulate cortex in addition to the insula and OFC. The two neuroimaging meta-analyses methods identified the caudate nucleus as a key area associated with satiety regulators. Our results provide quantitative brain activation maps of neurohormonal gut-brain signaling in heathy-weight adults that can be used to define alterations with eating behavior.

## Highlights

• First functional neuroimaging meta-analysis to generate neurohormonal gut-brain activation maps.
• Meta-analyses reveal satiety regulators modulate hypothalamus and caudate activity.
• ALE and ABC meta-analysis approaches show similar results for caudate association.
• Appetite regulators modulate insula, amygdala, hippocampus, OFC activities.
• Satiety regulators modulate hypothalamus, thalamus, caudate, insula, putamen, OFC.

## Graphical Abstract

**Figure:**
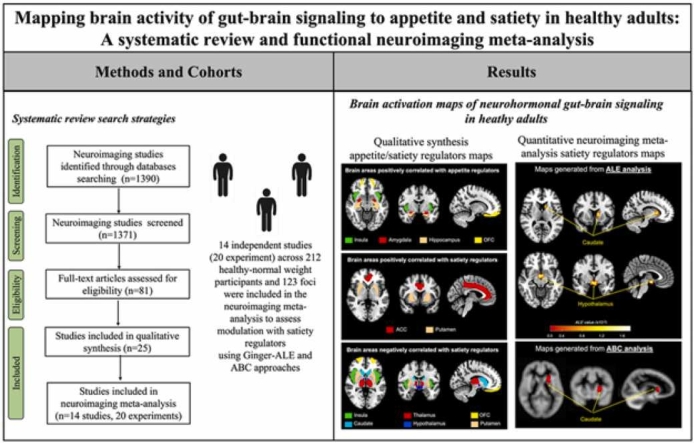

## 1 Introduction

The sensations of appetite and satiety are controlled by the central nervous system and involve complex interactions between appetite and satiety regulators and the brain. Clinical and preclinical studies show that an increase in endogenous ghrelin concentrations after fasting activates appetite-related neurons in the hypothalamus and stimulates appetite sensation and meal initiation (Cummings et al., 2001). Feeding reduces ghrelin concentrations and triggers the release of satiety regulators from the gastrointestinal tract, pancreas and adipose tissues. These regulators (cholecystokinin (CCK), glucagon-like peptide 1 (GLP-1), peptide YY (PYY), leptin and insulin) stimulate receptors in the vagus nerve and brain regions involved in the regulation and inhibition of food intake (Ahima and Antwi, 2008, Cummings and Overduin, 2007). Recent studies have shown that gut hormones can be manipulated to regulate food intake in humans (Malik et al., 2008) and may provide an effective and well-tolerated treatment for obesity and patients with altered appetite. For example, exogenous ghrelin administered to both lean and obese human volunteers has been shown to increase food intake (Druce et al., 2006, Druce et al., 2005). In contrast, PYY infusion reduces hunger and caloric intake in obese and lean subjects (Batterham et al., 2003), making exogenous PYY a potential therapy for obesity.

Recent advances in non-invasive neuroimaging techniques have allowed the study of neurohormonal gut-brain signaling pathways that modulate appetite in health and disease (Gibson et al., 2010). However, reported responses in brain areas associated with endogenously released appetite or satiety regulators, following ingestion of food, or exogenously administered hormones show inconsistency. There are several reasons for these discrepancies, including different study designs (endogenously released vs. exogenous administration) or different paradigms/stimulations (task-free “rest” vs task-based “i.e., food images or taste stimuli) used during brain imaging. However, by pooling data from published work on the interplay between appetite and satiety regulators and the brain in modulating appetite, we may establish a more accurate picture of regional brain activation associated with appetite and satiety processing. Coordinate-based neuroimaging meta-analyses including activation likelihood estimation (ALE) methodology (Turkeltaub et al., 2002) and Analysis of Brain Coordinates (ABC) (Tench et al., 2021) allow the identification of consistent brain activations across studies. These techniques use coordinates in standard anatomical space reported by neuroimaging studies to assess the agreement, or overlap, in activation patterns and infer a quantitative brain map of the overlapped regions (Muller et al., 2018, Tench et al., 2021).

The primary aim of this study was to provide a comprehensive analysis of the functional neuroimaging literature on brain areas associated with changes in appetite and satiety regulators in healthy-weight adults, and to produce a quantitative brain activation map of the neurohormonal gut-brain interactions, using coordinate-based neuroimaging meta-analysis. We hypothesize that the insula, hypothalamus, and caudate nucleus are commonly reported brain areas across studies.

## 2 Materials and methods

### 2.1 Systematic review of the literature

A comprehensive search was carried out in the MEDLINE, EMBASE and Cochrane Central Register of Controlled Trials (CENTRAL) databases between November 2019 and January 2021 to identify relevant studies using keywords from functional neuroimaging techniques and appetite and satiety responses including terms related to satiety and/or appetite regulators.

#### 2.1.1 Search strategy

The full search strategy is described in supplementary Table 1. This systematic review was reported in accordance with the Preferred Reporting Items for Systematic Reviews and Meta-Analyses (PRISMA) guidelines (Liberati et al., 2009). The protocol was registered on the International Prospective Register of Systematic Reviews (PROSPERO) (https://www.crd.york.ac.uk/prospero/) with registration number CRD42020223921. Searches were restricted to human studies published in the English language but not restricted to publication dates. After duplicates were removed, all records were screened for title and abstract. The remaining publications were then reviewed independently for eligibility based on full texts by three authors using the set criteria. Reviewers resolved discrepancies through discussion, or, if needed, by adjudication by a fourth reviewer. The eligibility criteria were based on the PICO (Population-Intervention-Comparator-Outcomes) model, summarized in Table 1.

**Table 1:** Eligibility criteria based on the PICO model.

**Inclusion criteria**	**Population**	• Healthy normal weight (18.5> BMI <25 kg/m^2^) human adults between 18 and 65 years old with no medications that would influence appetite or metabolism
	**Intervention**	• Acute macronutrients interventions (carbohydrate, protein or fat) consumed as a drink or a meal or • Exogenous infusion of appetite/satiety regulators • No restrictions were applied on the amount of macronutrients given, the level of hormone infusion, the number of hours fasted, the consumption/infusion and the route of macronutrient ingestion (oral or gastric) or gut hormone infusion (bolus, intravenous or subcutaneous injection)
	**Comparator**	• No specific comparators with controls such as water, placebo, saline or fasting included. Most studies are “before and after” intervention where the participants serve as their own controls
	**Outcomes**	• Primary outcome: concurrence of brain regions modulated in response to appetite and satiety regulators in healthy weight participants. • Secondary outcome: quantitative brain-activation maps generated from coordinate based meta-analyses to assess the concurrence of brain regions modulated in response to appetite and satiety regulators.
	**Study design**	• Controlled trials, randomized controlled trials, randomized cross-over design trials and cohort studies
**Exclusion criteria**		• Studies that involved participants with gastrointestinal, endocrine and neurological diseases, adolescents, overweight participants or those with obesity (BMI >30 kg/m^2^). For studies which combined data for healthy weight and participants with obesity, if possible, the data for the healthy weight participants were extracted • Publications with no direct correlation analysis performed between brain responses and satiety/appetite regulators or with long-intervention studies • In-vitro studies • Reviews

#### 2.1.2 Risk-of-bias assessment

The quality of the included papers was assessed for potential risk of bias by one author (SA), using the Cochrane collaboration to assess the risk of bias in randomized cross-over and randomized controlled trials (ROBINS-I) (Ding et al., 2015, Higgins et al., 2019). The quality assessment of each paper is shown in Supplementary Table 2.

#### 2.1.3 Data extraction

For each study, the following information was extracted: authors, year of publication, total number of participants, participant details [mean age, sex and body mass index (BMI)], time of first brain imaging scan after treatment administration, intervention, administration method (e.g. oral, gastric or intravenous ), assessed appetite and/or satiety regulators that was directly correlated with brain responses, neuroimaging modality and brain stimulation method (e.g. gustatory, visual) and correlation results between brain areas and appetite and satiety regulators. Extracted data were grouped into 1) brain areas correlated positively 2) and/or correlated negatively with appetite regulators, 3) brain areas correlated positively and/or 4) correlated negatively with satiety regulators. In the appetite analysis, data were analyzed during the fasting state or for contrast fasted>fed. In the satiety state analysis, data were derived from a direct contrast between fed state versus fasted/hunger state (fed>fasted) or data assessed postprandially within 1.5 h following the last consumption. Brain areas from each of the sub-grouped data were then pooled and common brain areas across studies were evaluated. To illustrate the concurrence of brain areas generated from the systematic review, anatomically defined masks for the overlapped brain areas were generated using WFU PickAtlas toolbox (Maldjian et al., 2003) in SPM software (https://www.fil.ion.ucl.ac.uk/spm/software/spm12/). The generated masks were displayed using the MRIcroGL software (Rorden et al., 2007) and overlaid on brain template in MNI space.

### 2.2 Coordinate-based neuroimaging meta-analysis

Neuroimaging studies included in the meta-analysis were pooled from those included in the systematic review. Studies that did not report coordinates for brain activations in response to appetite/satiety regulators in the article or supplementary material were excluded from the meta-analysis. We followed the recently suggested standard protocols of neuroimaging meta-analysis by Eickhoff et al. (2016) and included only neuroimaging studies that reported brain activation using whole-brain voxel wise analyses (Turkeltaub et al., 2012). In addition, seed-based functional connectivity analysis in rs-fMRI studies were excluded from the meta-analysis, as they usually focus on particular areas in the brain. Coordinates of brain regions that are directly correlated with satiety and appetite regulators were manually extracted by two authors (SA and SE), independently. Extracted coordinates were checked and when inconsistencies between the coordinates reported in the original study were identified coordinates were rechecked and corrected. Studies that reported coordinates in Talaraich space (Tournoux and Talariach, 1988) were converted to the standard space of the Montreal Neurological Institute (MNI) (Evans et al., 1993) using the icbm2taal algorithm implemented in the Ginger ALE toolbox (Laird et al., 2010).

There are multiple algorithms for performing coordinate-based neuroimaging meta-analysis, each has different empirical parameters and assumptions, and each can produce different results conditional on the assumptions. Therefore, to obtain robust results of brain areas associated with appetite and satiety regulators, two different tools were employed: the ALE and ABC meta-analysis methods. The ALE-approach is the most popular method of performing neuroimaging meta-analysis. The algorithm takes into account the number of participants in each study to apply a relevant smoothing, resulting in a higher specificity of the actual overlap between studies (Eickhoff et al., 2009). However, in order to produce results that are not overly representative of single studies, it is recommended that at least 17–20 experiments should be included in the analysis. In addition, in contrast to the ABC-approach, the ALE algorithm does not allow assessment of the effect sign associated with the coordinates when a decrease/increase brain activity are combined in a single analysis. Furthermore, ABC requires a minimum of only 5 studies, but in doing so it does not take any account of the study the sample size (the number of participants in each study). A further difference in between the two algorithms is in the thresholding for statistical significance, where ALE uses a cluster level family wise error rate method, ABC directly relates the threshold to the aim of detecting replicated results.

#### 2.2.1 Activation likelihood estimation (ALE) analysis

ALE meta-analyses were performed using Ginger ALE version 3.0.2 (http://www.brainmap.org/ale). ALE analysis uses the reported activation peaks from the individual studies as a three-dimensional Gaussian probability distribution (kernel) centered at the given coordinates to create a modeled activation (MA) map for each study. Individual MA-maps are then combined to calculate statistical ALE maps and ALE values for each cluster. These calculations are confined to a grey matter mask provided by the Ginger ALE software. The ALE maps indicate areas of the brain where convergence between activation foci is greater than would be expected by chance (i.e., a null distribution of clusters). We adhered to the recommendations of Eickhoff et al. (2016) for all analyses. The statistical significance of ALE maps was assessed and corrected for multiple comparisons by employing a cluster-level family-wise error (FWE) at P < 0.05, following an initial cluster forming threshold of uncorrected P < 0.001 (Eickhoff et al., 2016). The P-value was calculated for each voxel based on probabilities of reaching an ALE value that differed from that of the corresponding voxel on a null-distribution map, via random permutation. We used 5000 permutations to generate the P-values (Laird et al., 2010). The generated meta-analysis maps from the ALE methods were displayed using the MRIcroGL software (Rorden et al., 2007) and overlaid on brain template in MNI space.

#### 2.2.2 Analysis of brain coordinates (ABC)

ABC methodology (Tench et al., 2021) was performed using the ABC toolbox implemented in the NeuRoi image analysis software (https://www.nottingham.ac.uk/research/groups/clinicalneurology/neuroi.aspx). The algorithm of this recently developed model-based method uses the density of coordinates from independent studies as its statistic and requires only the human grey matter volume (one parameter). Statistical thresholding is performed by requiring a minimum proportion of the studies contributing to a cluster and is generally more conservative than false discovery rate (FDR < 0.05). Importantly, this method, in contrast to the ALE-approach, does not require the empirical choice of Gaussian smoothing kernel to extrapolate coordinates to voxel-wise activation maps or the randomization of the coordinates in the empirical space to define the statistical threshold.

## 3 Results

### 3.1 Systematic review

#### 3.1.1 Selection and inclusion of studies

**Fig. 1:**
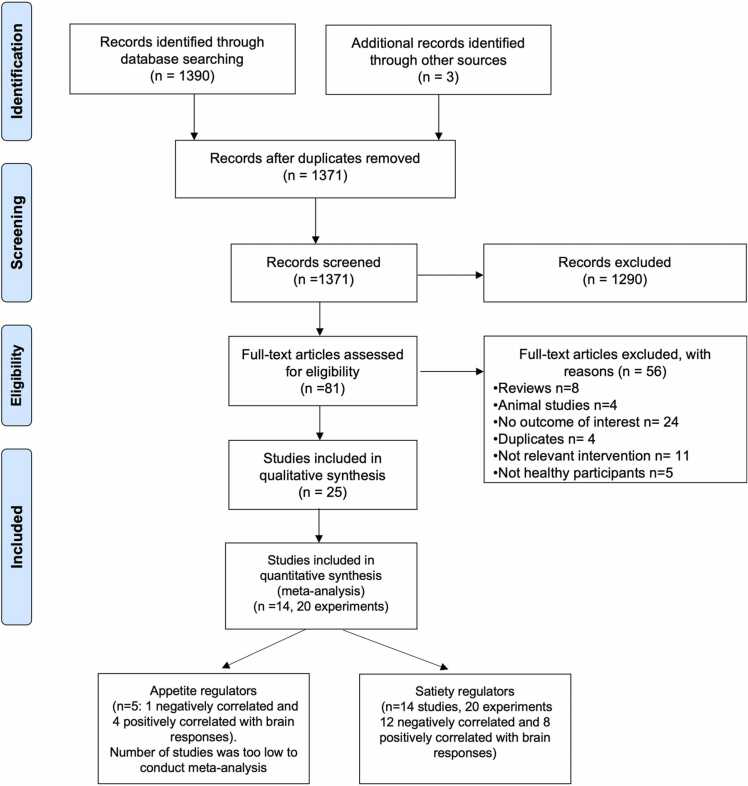
PRISMA Diagram.

Of the 1390 studies identified in the initial search, 81 were selected for full text assessment. Eligibility criteria were based on the PICO (Population-Intervention-Comparator-Outcomes) model, and the inclusion and exclusion criteria are listed in Table 1. A total of 25 eligible studies were included (see Table 2) for full data extraction (see PRISMA diagram, Fig. 1).

**Table 2:** Characteristics and main results of the included studies in the systematic review and coordinate based meta-analysis.

Authors and year of publication	Sample size	Mean age (years ± SD), Sex & BMI (Kg/m^2^)	Intervention & time of intervention	Brain region investigated	Administration	Appetite/satiety regulators investigated	Neuroimaging modality & paradigm	Results
Al-Zubaidi et al. (2019)	n= 24	• Age: 24.3±1.3 • Sex: all male • BMI: 24.4 ± 1.4	• 300 ml of glucose (75 g) ingestion • Fasted state • 20 min after meal ingestion	Whole brain	Orally	• Glucose • Insulin	rs-fMRI	After glucose ingestion relative to fasting (hunger > satiety): • insulin levels: superior frontal gyrus ↓, posterior insula ↓ • glucose: fusiform gyrus ↑
Batterham et al. (2007) *	n=8	• Age: 29.6 ± 2.1 • Sex: all male • BMI: 21.7 ± 0.7	• PYY infusion • Placebo (saline) infusion • Immediately after infusion	Whole brain + ROI (solitary nucleus and tract, parabrachial nucleus, substantia nigra, nucleus accumbens & hypothalamus)	Intravenous	• Glucose • Insulin • PYY • Ghrelin	Physiological fMRI	After PYY infusion: • ghrelin: hypothalamus ↑, VTA ↑ & brainstem ↑ • PYY: globus pallidus ↑, middle frontal gyrus ↑, anterior lobe cerebellum ↑, anterior cingulate ↑, inferior parietal lobule ↑, medial superior frontal gyrus ↑, substantia nigra ↑, OFC ↑, peri-aqueductal grey ↑, VTA ↑, precentral gyrus ↑, parabrachial nucleus ↑, insula ↑, putamen ↑, hypothalamus ↑, superior temporal gyrus ↑, middle frontal gyrus ↓, angular gyrus ↓
De Silva et al. (2011)	n=16	• Age: 29.5 • Sex: 11 male & 5 female • BMI: 22.1	• Saline infusion • Standard breakfast (579 kcal), then saline infusion, • 0.8 pmol/kg/min of GLP-17-36 amide • 0.3 pmol/kg/min of PYY 3-36 • Combined PYY3-36 & GLP-17-36 amide &(0.3 pmol/kg/min & 0.8 pmol/kg/min respectively) • 90 min after infusion	ROI (bilaterally amygdala insula, OFC, nucleus accumbens, caudate & putamen)	• Orally for the breakfast • Intravenous for hormones infusion	• GLP-1 • PYY • Combined GLP-1 and PYY	task-fMRI- (food picture paradigm)	• PYY: OFC ↓, nucleus accumbens ↓ • GLP-1: insula ↓
Dorton et al. (2017)	n= 22	• Age: 21.2 ± 2.1 • Sex: 10 male & 12 female • BMI: 22.6 ± 1.9	• 300 ml of glucose (75 g) ingestion • 300 ml of water ingestion • 20 min after meal ingestion	ROI [ventral striatum (nucleus accumbens) and bilateral dorsal striatum (caudate/putamen)]	Orally	• GLP-1 • PYY	task-fMRI (food picture paradigm)	After glucose ingestion: • GLP-1: dorsal striatal ↓
Eldeghaidy et al. (2016) *	n= 17	• Age: 25 ± 2 • Sex:11 male & 6 female • BMI: 22.4 ± 0.8	Two emulsion stimuli; flavored fat stimulus (FS) & flavored not fat control stimulus (CS) following: • 250 ml of high fat drink/load (22% fat) • 250 ml of water load • 45 min after meal ingestion	Whole brain	Orally	• CCK	task-fMRI (taste stimuli paradigm)	Responses to the CS and FS after the high fat drink/load: • CCK: primary somatosensory cortex ↓, amygdala↓, supramarginal gyrus ↓, middle and posterior insula ↓, temporal gyrus ↓, thalamus ↓, cerebellum ↓, operculum ↓
Gautier et al. (2000) *	n= 11	• Age: 35 ±8 • Sex: all male • BMI: 25	• Liquid formula meal (1.5 kcal/ml Ensure-Plus: 15% protein, 53% carb & 32% fat) • 25 min after meal ingestion	Whole brain + ROI (hypothalamus, thalamus, DLPFC, anterior prefrontal cortex, ACC, insular cortex, posterior orbitofrontal cortex, hippocampus/parahippocampal gyrus, caudate ventricle, precuneus, putamen, parietotemporal cortex, occipital cortex, cerebellum & midbrain)	Orally	• Insulin • GLP-1 • Leptin • FFA	PET	After the liquid meal ingestion: • Insulin: posterior OFC↓, hippocampus/ parahippocampus ↓, putamen ↓, thalamus ↓, precuneus ↑ • FFA: DLPFC ↑
Goldstone et al. (2014)	n= 22	• Age: 25.9 ± 1.7 • Sex: 17 male & 5 female • BMI: 23.9 ± 0.6	• Fasted saline injection • Fed saline injection with standard breakfast (730 kcal, 55% CHO, 31% fat & 14% protein) • Fed ghrelin injection with standard breakfast (730 kcal, 55% CHO, 31% fat & 14% protein) • 95 min after infusion	Whole brain + ROI (nucleus accumbens, caudate, anterior insula, amygdala, hippocampus, OFC)	Intravenous	• Glucose • Insulin • GLP-1 • PYY • Ghrelin • TG	task-fMRI (food picture paradigm)	• ghrelin: OFC ↑, hippocampus↑
Heni et al. (2015) *	n=12	• Age: 23±2 • Sex: 6 male & 6 female • BMI: 21.1 ± 1.1	• 300 ml of glucose (75 g) ingestion • 300 ml of water ingestion • 30 min after meal ingestion	Whole brain	Orally	• GLP-1	task-fMRI (food picture paradigm)	After glucose ingestion: • insulin: OFC ↓
Jakobsdottir et al. (2012) *	n= 15	• Age: 23.4 ± 3.5 • Sex: all male • BMI: 22.4 ± 2	• Standard meal consisted of 1600 kcal, 15.8 % protein, 44.4% carbohydrate and 39.8% fat • 60 min after meal ingestion	Whole brain	Orally	• Glucose • Insulin • Ghrelin • TAG • Leptin	task-fMRI (food picture paradigm)	After satiation with standard meal: • leptin: hippocampus ↓, insula ↓, temporal lobe bilaterally ↓, frontal gyrus ↓
Jones et al. (2012)	n= 20	• Age: 34.1 • Sex: 7 male & 5 female • BMI: 25.1	• Fasting state: 1- ghrelin injection (1.25 or 5 pmol/kg/min) 2- intragastric lipid (dodecanoate, C12) + ghrelin • Postprandial state: 1- ghrelin bolus (0.3 mmol/kg) 2- saline • Immediately after infusion	Whole brain	Intravenous	• Ghrelin	Physiological fMRI	• Ghrelin (pre-prandial/fasting state): thalamus ↑, hypothalamus ↑, midbrain, cerebellum↑, medulla ↑, pons ↑, mid-brain ↑, amygdala ↑, hippocampus ↑, insula↑, precentral gyrus ↑, postcentral gyrus ↑ • Ghrelin (post-prandial state): thalamus ↓, amygdala ↓ hippocampus ↓, insula ↓, hypothalamus ↓, midbrain ↓, pons ↓, medulla ↓, postcentral gyrus ↓, cerebellum↓, precentral gyrus ↑ motor cortex ↑
Kroemer et al. (2013)	n=26	• Age: 24.4 ± 3.4 • Sex: 13 male & 13 female • BMI: 21.1 ± 2	• 300 ml of glucose (75 g) ingestion • 5 min after meal ingestion	Whole brain	Orally	• Ghrelin	task-fMRI (food picture paradigm)	• Fasting ghrelin: middle frontal gyrus ↑, midbrain ↑**,** superior/medial frontal gyrus ↑**,** inferior frontal gyrus ↑**,** medial occipital/temporal gyrus ↑**,** hypothalamus ↑, subthalamic nucleus ↑**,** fusiform gyrus ↑**,** thalamus ↑**,** superior occipital gyrus, inferior frontal gyrus ↑**,** middle frontal gyrus ↑**,** pallidum, amygdala ↑**,** inferior frontal gyrus, caudate body ↑**,** inferior temporal g., fusiform gyrus ↑**,** middle/superior frontal gyrus ↑**,** thalamus (anterior nucleus) ↑**,** medial/superior frontal gyrus, ↑anterior cingulate ↑, postcentral (supramarginal gyrus & rolandic operculum ↑)
Lassman et al. (2010) *	n=19	• Age: 37 • Sex: male & 6 female • BMI: 25.4	• 250 ml lipid (dodecanoic acid) • 250 ml of saline (0.9% control) • CCK receptor antagonist dexloxiglumide (600 mg), administrated orally 1 hour before the intragastric infusion • Immediately after infusion	Whole brain	Intragastric infusion	• CCK	Physiological fMRI	• CCK: hypothalamus ↓, medulla ↓, midbrain ↓, precuneus ↓, cerebellum ↓, cingulate gyrus ↓, caudate ↓, thalamus ↓, temporal gyrus ↓
Li et al. (2012)	n= 14	• Age: 23 • Sex: all male • BMI: 21.2	• 300 ml of whey • protein (257 g/L) ingestion • 300 ml of soybean emulsion (111 g/L) ingestion • 300 ml of glucose (250 g/L) ingestion • 300 ml of water ingestion • 6 min after meal ingestion	ROIs (thalamus, hypothalamus, insula, parahippocampal/hippocampal cortex, putamen, caudate OFC & amygdala)	Orally	• GLP-1 • Ghrelin • Glucose • Insulin • CCK	task-fMRI (taste stimuli paradigm)	After whey protein ingestion: • GLP-1: Lateral orbito-frontal cortex↓ • insulin: Caudate↓ • CCK: thalamus ↓ • ghrelin: amygdala ↑ After fat ingestion: • CCK: caudate, thalamus ↓ • ghrelin: amygdala ↑, middle insula ↑, lateral OFC ↑ After glucose ingestion: • insulin: thalamus↓, middle insula↓, amygdala↓, lateral OFC ↓ • glucose: thalamus↓ • CCK: caudate ↓ • GLP-1: latera OFC ↓, middle insula↓ • ghrelin: middle insula ↑, later OFC ↑
Liu et al. (2000)	n= 21	• Age: 34 ± 3 • Sex: 11 male & 10 female • BMI: NA	• 296 ml of dextrose (75 g) ingestion • 300 ml of distilled water ingestion • 10 min after meal ingestion	Whole brain	Orally	• Insulin	rs-fMRI	• Fasting insulin: hypothalamus ↓, somatosensory cortex ↓, SMA ↓, cerebellum↓, anterior cingulate ↓, OFC ↓
Little et al. (2014) *	n=12	• Age: 38 ± 3.4 • Sex: 7 male & 5 female • BMI: 19.7 - 28.9	• 250 ml of glucose (45g) following 2 placebo tablets • 250 ml of glucose (45g) following 600 mg of CCK1 receptor antagonist (dexloxiglumid) • 250 ml of saline (0.9%, control) following 2 placebo tablets • 60 min after infusion	Whole brain	Intravenous& intragastric infusion	• Glucose • Insulin • CCK • GLP-1	Physiological fMRI	Glucose vs saline: • glucose: hypothalamus↓, brainstem ↓, medulla↓, pons↓, cerebellum↓ cerebellum anterior ↓, lingual ↓, fusiform↓, thalamus↓ • insulin: hypothalamus↓, brainstem ↓, medulla↓, pons↓, cerebellum↓ cerebellum anterior ↓, lingual ↓, fusiform↓, thalamus↓. Glucose + dexloxiglumide vs saline: • Insulin: cerebellum↓, lingual gyrus ↓, cuneus ↓ • GLP-1: cerebellum ↓, lingual gyrus ↓, cuneus ↓
Malik et al. (2008)	n=21	• Age: 24.1 ± 1.1 • Sex: all male • BMI: 22.3 ± 0.7	• Ghrelin infusion • Placebo (saline) infusion. • Immediately after infusion	Whole brain	Intravenous	• Ghrelin	task-fMRI (food picture paradigm)	• After ghrelin infusion: hippocampus ↑, amygdala ↑, OFC ↑, caudate ↑, pulvinar ↑, VTA ↑, substantia nigra ↑, insula ↑, occipital gyrus↑, fusiform ↑
Page et al. (2009) *	n= 9	• Age: 28 ± 5 • Sex: 8 male & 1 female • BMI: 23.6 ± 2	• Euglycemia (2 mU/kg/ min of insulin+ 20 % glucose adjusted to achieve euglycemia (plasma glucose= 95 mg/dL) • Hypoglycemia (plasma glucose= 50 mg/dL) • 30 min after hypoglycemic. • 90 min after euglycemic. • Immediately after infusion	Whole brain + ROI (hypothalamus)	Intravenous	• Glucose • Insulin	fMRI-ASL	• Hypoglycemia relative to euglycemia (hypoglycemia > euglycemia): hypothalamus↑, inferior frontal gyrus ↑, ACC ↑, caudate↑, pars triangularis L ↑, superior temporal gyrus ↑, visual association cortex ↑, putamen ↑, pars opercularis ↓, medial frontal gyrus↓, cerebellum↓,
Page et al. (2011) *	n= 21	• Age: 31.4 ± 7.9 • Sex: 12 male & 9 female • BMI: 25.2 ± 4	• Euglycemia (2 mU/kg/ min of insulin+ 20 % glucose adjusted to achieve euglycemia (plasma glucose= 95 mg/dL) • Hypoglycemia (plasma glucose= 50 mg/dL) • Immediately after infusion	Whole brain	Intravenous	• Insulin • Ghrelin • Leptin	task-fMRI (food picture paradigm)	• Euglycemia relative to hypoglycemia (euglycemia > hypoglycemia): anterior cingulate cortex↑, ventromedial-prefrontal cortex ↑
Page et al. (2013) *	n= 20	• Age: 31 ± 7 • Sex: 10 male & 10 female • BMI: 22 ± 2.5	• 300 ml of glucose (75 g) ingestion • 300 ml of fructose (75 g) ingestion • 60 min after meal ingestion	Whole brain + ROI (hypothalamus)	Orally	• GLP-1 • PYY • Ghrelin	rs-fMRI & fMRI-ASL	After glucose ingestion: • insulin: caudate ↓, putamen ↓
Pannacciulli et al. (2007) * ♯	n= 42	• Age: 31 ± 8 • Sex: 22 male & 20 female • BMI: 31 ± 9	• Standard liquid formula meal (1.5 kcal/ml Ensure-plus, 15% protein, 53% carbohydrate and 32% fat) • 25 min after meal ingestion	Whole brain	Orally	• GLP-1	PET	After the liquid meal ingestion: • GLP-1: hypothalamus ↑, inferior frontal gyrus ↑, middle frontal gyrus ↑
Schilling et al. (2014)	n=48	• Age: 23.96 ± 3.4 • Sex: all male • BMI: 20 < BMI > 25	• Oral cortisol vs. intranasal insulin • Oral cortisol vs. oral placebo • Oral vs. intranasal placebo. • Intranasal insulin vs. intranasal placebo • Insulin (100 I.E. /ml) & cortisol (30 mg) • 30 min after infusion	Whole brain + ROI (hippocampus, insula, putamen)	Intravenous	• GLP-1 • Insulin	fMRI-ASL	• Intranasal insulin infusion: putamen ↑, insula↑, inferior frontal gyrus↑, caudate nucleus↑
Spetter et al. (2014) *	n= 14	• Age: 24.6± 3.8 • Sex: all male • BMI: 22.3 ± 1.6	• Oral chocolate milk • nasogastric water • nasogastric chocolate milk infusion (per 100ml; 84.6 kcal, 16% protein, 56.7% carbohydrate and 26% fat) • 5 min after meal ingestion	Whole brain + ROI (hippocampus insula, amygdala, midbrain, putamen, caudate, pallidum, nucleus accumbens and hypothalamus)	Orally & Nasogastric tube	• Glucose • Insulin • Ghrelin • CCK	task-fMRI (taste stimuli paradigm)	During nasogastric infusion of chocolate milk: • Insulin: middle and posterior Insula ↓, putamen↑
Sun et al. (2014)	n= 32	• Age: 25.3 ± 5.6 • Sex: 15 male & 17 female • BMI: 25.3 ± 4.5	Milkshake (per 945 ml; 918 kcal, 10.7% protein, 52.4% carbohydrate and 25% fat) during: • Fasting • Satiation with fixed lunch meal (425 kcal for women & 625 kcal for men) and satiation with ad lib lunch meal • 30 min after meal ingestion	ROI (hippocampus insula, amygdala, midbrain, putamen, caudate, pallidum, nucleus accumbens and hypothalamus)	Orally	• Glucose • Insulin • Ghrelin • TAG	task-fMRI (taste stimuli paradigm)	Responses to milkshake after the fixed meal: • ghrelin: amygdala↑, midbrain↑, insula↑, pallidum↑, hippocampus↑ • TAG: midbrain ↓, insula ↓, hippocampus ↓, putamen ↓, pallidum ↓
Tataranni et al. (1999) *	n= 11	• Age: 34 ± 3 • Sex: all male • BMI: 19 ± 6 % body fat	• Liquid formula meal (1.5 kcal/ml Ensure-Plus: 15% protein, 53% carb & 32% fat) • 25 min after meal ingestion	Whole brain	Orally	• Glucose • Insulin • Leptin • FFA	PET	After the liquid meal ingestion: • Insulin: OFC↓, insula ↓ • FFA→ OFC↓, insula ↓, DLPFC ↑
Wolnerhanssen et al. (2015) *	n= 12	• Age: 24.8 • Sex: all male • BMI: 22.9	• 300 ml of glucose (75 g) ingestion • 300 ml of fructose (75 g) ingestion • 300 ml of placebo (water) of ingestion • 5 min after meal ingestion	Whole brain	Nasogastric tube	• Insulin • Glucose • GLP-1	rs-fMRI	After glucose ingestion relative to placebo (glucose > placebo): • insulin: caudate ↑, pallidum ↑, OFC ↑

ACC, anterior cingulate cortex; ASL, arterial spin labelling; CBF, cerebral blood flow; CCK, cholecystokinin; DLPFC, Dorsolateral prefrontal cortex; FFA, free fatty acids; fMRI, functional magnetic resonance imaging; GLP-1, Glucagon-like peptide-1; OFC, orbitofrontal cortex; PET, position emission tomography; PYY, peptide YY; rs-fMRI, resting state fMRI; ROI, region of interest; SMA, supplementary motor area; VTA, ventral tegmental area. “↓” indicates decreased brain activation and “↑” indicates increased brain activation. Asterisk “*” indicates studies included in the coordinate-based neuroimaging meta-analysis and # indicates a study included in the systematic review and coordinate based meta-analysis with BMI > 30 kg/m2. The effects of obesity on neuronal activity was accounted in this study as BMI was included as a covariate of no interest in the fMRI analysis.

#### 3.1.2 Characteristics of included studies

Of the 25 studies included, twenty-two studies used functional magnetic resonance imaging (fMRI), of which eight used food picture task-fMRI (De Silva et al., 2011, Dorton et al., 2017, Goldstone et al., 2014, Heni et al., 2015, Jakobsdottir et al., 2012, Kroemer et al., 2013, Malik et al., 2008, Page et al., 2011), five studies used taste stimuli (Eldeghaidy et al., 2016, Li et al., 2012, Liu et al., 2000, Spetter et al., 2014, Sun et al., 2014), four studies assessed neurological responses across a time course “physiological fMRI design”(Batterham et al., 2007, Jones et al., 2012, Lassman et al., 2010, Little et al., 2014), three studies used resting state fMRI (Al-Zubaidi et al., 2019, Page et al., 2013, Wolnerhanssen et al., 2015), two studies used arterial spin labelling (ASL) (Page et al., 2009, Schilling et al., 2014). Three studies used position emission tomography (PET) imaging technique (Gautier et al., 2000, Pannacciulli et al., 2007, Tataranni et al., 1999).

Across the included studies, brain responses for the hungry state were assessed following fasting that ranged between 4 and 14 h, whereas for the satiety state brain responses were assessed within 1.5 h postprandially. Seven studies administered standard meals with different amounts of protein, fat and fiber, containing ingredients such as soya bean, beef or milkshake (Al-Zubaidi et al., 2019, Gautier et al., 2000, Jakobsdottir et al., 2012, Pannacciulli et al., 2007, Spetter et al., 2014, Sun et al., 2014, Tataranni et al., 1999). Eight studies administered target nutrients such as whey protein solution, glucose drink, soybean oil emulsion or flavored fat emulsion samples (Dorton et al., 2017, Eldeghaidy et al., 2016, Heni et al., 2015, Kroemer et al., 2013, Li et al., 2012, Liu et al., 2000, Page et al., 2013, Wolnerhanssen et al., 2015). Fifteen studies reported that nutrients were administered orally (Al-Zubaidi et al., 2019, De Silva et al., 2011, Dorton et al., 2017, Eldeghaidy et al., 2016, Gautier et al., 2000, Heni et al., 2015, Jakobsdottir et al., 2012, Kroemer et al., 2013, Li et al., 2012, Liu et al., 2000, Page et al., 2013, Pannacciulli et al., 2007, Spetter et al., 2014, Sun et al., 2014, Tataranni et al., 1999), while three studies administered nutrients via the intra-gastric route (Little et al., 2014, Spetter et al., 2014, Wolnerhanssen et al., 2015). Spetter et al. (2014) reported that they administered nutrients by both the nasogastric tube and the oral routes. Ten studies administered exogenous appetite and satiety regulators including PYY, GLP-1, ghrelin, insulin, glucose and CCK by intravenous (Batterham et al., 2007, De Silva et al., 2011, Goldstone et al., 2014, Jones et al., 2012, Malik et al., 2008, Page et al., 2009, Page et al., 2011), intranasal (Schilling et al., 2014) or intra-gastric infusion (Lassman et al., 2010, Little et al., 2014). Details of the included studies are provided in Table 2.

#### 3.1.3 Modulation of brain responses to appetite and satiety regulators

**Fig. 2:**
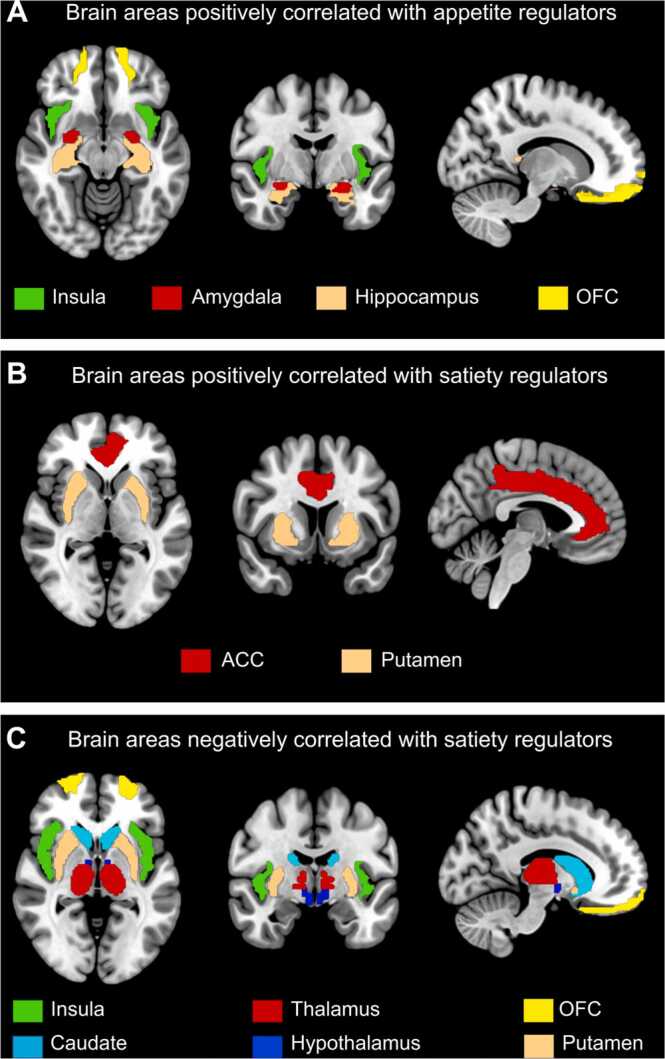
Results of the systemic review showing concurrence of key brain areas commonly reported across studies. **(A)** Brain areas positively correlated with appetite regulators, showing the concurrence in the insula, amygdala, hippocampus, and orbitofrontal cortex (OFC). **(B)** Brain areas positively correlated with satiety regulators showing the concurrence in the anterior cingulate gyrus (ACC) and the putamen. **(C)** Brain areas negatively correlated with satiety regulators showing the concurrence in the insula, caudate, thalamus, hypothalamus, OFC, and putamen.

As a first step, the data extracted from the 25 studies were grouped into brain areas that correlated: 1) positively and/or 2) negatively with appetite regulators, 3) correlated positively and/or 4) negatively with satiety regulators. Data from brain areas from each of the sub-groups were then pooled and common brain areas across studies evaluated. A full list of overlapped brain areas is reported in supplementary Table 3. The concurrence of key brain areas commonly reported across studies based on the findings from the systematic review is illustrated in Fig. 2.

##### 3.1.3.1 Appetite regulators

Eight studies (Batterham et al., 2007, De Silva et al., 2011, Goldstone et al., 2014, Jones et al., 2012, Kroemer et al., 2013, Li et al., 2012, Malik et al., 2008, Sun et al., 2014) reported positive correlation of ghrelin with concurrence in brain activation mostly found in the amygdala (five studies) (Jones et al., 2012, Kroemer et al., 2013, Li et al., 2012, Malik et al., 2008, Sun et al., 2014), orbitofrontal cortex (OFC) (five studies) (De Silva et al., 2011, Goldstone et al., 2014, Li et al., 2012, Malik et al., 2008, Sun et al., 2014), insula (four studies) (Jones et al., 2012, Li et al., 2012, Malik et al., 2008, Sun et al., 2014), and hippocampus (four studies) (De Silva et al., 2011, Goldstone et al., 2014, Jones et al., 2012, Malik et al., 2008), Fig. 2A. A single study reported negative correlation with ghrelin concentrations in the caudate nucleus, hypothalamus, insula, amygdala, hippocampus, and thalamus (Jones et al., 2012), therfore brain areas are not shown in Fig. 2.

##### 3.1.3.2 Satiety regulators

In terms of satiety regulation, eight studies reported positive modulation in response to satiety regulators with concurrence in the anterior cingulate cortex (ACC) (three studies) (Batterham et al., 2007, Page et al., 2009, Page et al., 2011) and putamen (three studies) (Batterham et al., 2007, Page et al., 2009, Schilling et al., 2014), Fig. 2B. Fifteen studies reported attenuation in activity in various brain areas (Al-Zubaidi et al., 2019, De Silva et al., 2011, Eldeghaidy et al., 2016, Gautier et al., 2000, Heni et al., 2015, Jakobsdottir et al., 2012, Lassman et al., 2010, Li et al., 2012, Liu et al., 2000, Page et al., 2009, Page et al., 2013, Schilling et al., 2014, Spetter et al., 2014, Sun et al., 2014, Tataranni et al., 1999). Most studies showed concurrence in the insula (eight studies) (Al-Zubaidi et al., 2019, De Silva et al., 2011, Eldeghaidy et al., 2016, Jakobsdottir et al., 2012, Li et al., 2012, Schilling et al., 2014, Spetter et al., 2014, Sun et al., 2014), hypothalamus (five studies) (Lassman et al., 2010, Liu et al., 2000, Page et al., 2009, Page et al., 2013, Spetter et al., 2014), OFC (four studies) (De Silva et al., 2011, Gautier et al., 2000, Heni et al., 2015, Li et al., 2012), thalamus (four studies) (Eldeghaidy et al., 2016, Gautier et al., 2000, Lassman et al., 2010, Li et al., 2012), putamen (four studies) (Gautier et al., 2000, Page et al., 2013, Spetter et al., 2014, Sun et al., 2014), and caudate nucleus (three studies) (Lassman et al., 2010, Li et al., 2012, Page et al., 2013), Fig. 2C.

### 3.2 Coordinate based meta-analysis

In a second step, we examined the concurrence/overlap in brain regions activated in response to changes in appetite and satiety regulators quantitatively using neuroimaging meta-analysis. Studies were initially grouped following the same methods used for the systemic review: 1) brain areas correlated positively with appetite regulators (4 studies), 2) and/or correlated negatively with appetite regulators (1 study), 3) brain areas correlated positively with satiety regulators (8 studies), 4) and/or correlated negatively with satiety regulators (12 studies). However, due to the small number of studies (less than the 17 required for the ALE analysis), for each of these the sub-groups, we could not perform separate meta-analysis. Instead, we performed two primary meta-analyses: one for appetite regulators and the other for satiety regulators, each combined across studies reported negative and positive correlation with brain responses.

#### 3.2.1 Concurrence of brain area modulated by appetite regulators

Of the five studies eligible for the meta-analysis with appetite regulators, four assessed positive correlation (Kroemer et al., 2013, Goldstone et al., 2014, Jones et al., 2012, Malik et al., 2008), and a single study assessed negative correlation (Jones et al., 2012). Due to the low number of investigations, this analysis was not performed.

#### 3.2.2 Concurrence of brain area modulated by satiety regulators

**Fig. 3:**
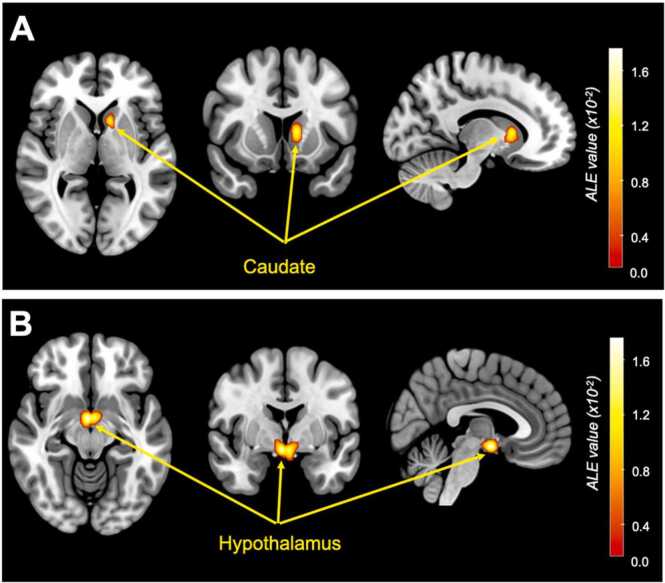
Results of the ALE meta-analysis showing convergent clusters with significant ALE values correlated with satiety regulators showing correlation in (A) the caudate nucleus centered at MNI (−10,12,6), Z = 4.62, ALE value= 1.5 × 10^-2^, cluster volume= 1000 mm^3^, and (B) the hypothalamus centered at MNI (2, −4,−12), Z = 4.21, ALE value= 1.32 × 10^-2^, cluster volume= 1728 mm^3^. Maps are family-wise error (FWE)-corrected for multiple comparisons P < 0.05.

**Fig. 4:**
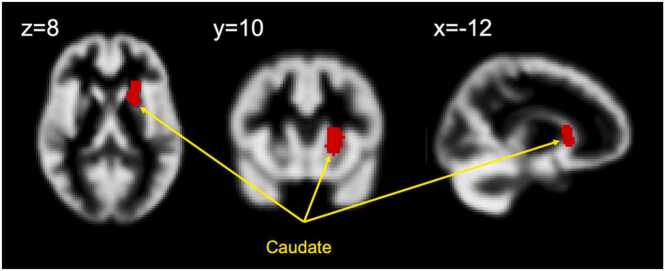
Results of the ABC meta-analysis, showing convergent clusters in the caudate nucleus centered at MNI (−12, 10, 8), False Discovery Rate (FDR) corrected for multiple comparisons < 0.05.

**Fig. 5:**
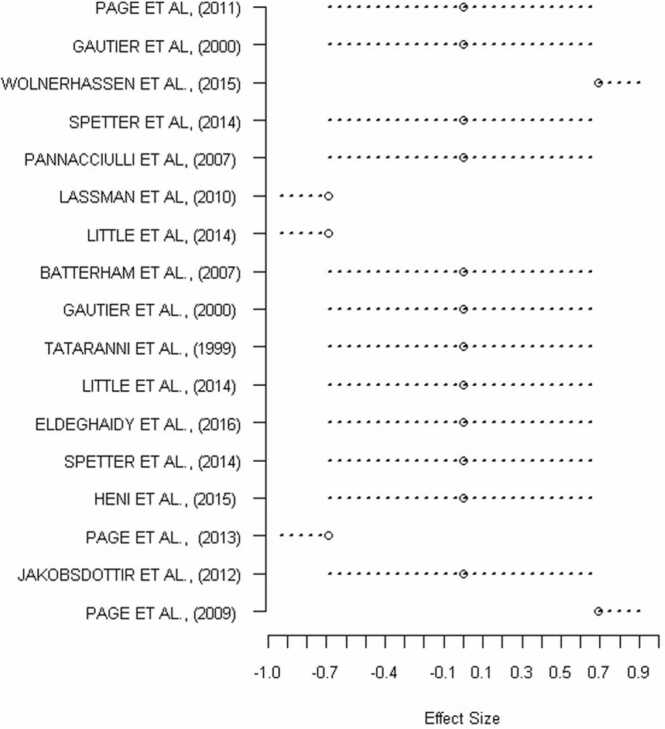
The forest plot from the ABC analysis illustrating the effect sign associated with studies contributing to the increased and/or decreased caudate activity in response to satiety regulators.

In terms of the satiety analysis, the ALE and ABC meta-analyses across 14 independent studies (20 experiments combined across increased/decreased brain activation to satiety regulators) included 212 healthy-weight participants and 123 foci provided convergent results, revealing the same cluster (see Fig. 3, Fig. 4) in the caudate nucleus. For the ALE-analysis, the caudate cluster was centered at MNI (−10,12,6) and for the ABC analysis at MNI (−12,10,8). Four studies contributed to the caudate cluster in the ALE-analysis, while five studies contributed to the ABC-analysis, see Table 3. The forest plot of the ABC approach (Fig. 5) demonstrated that two studies reported positive correlation (increase in caudate activity) while three studies reported negative correlation (decrease in caudate activity) with satiety regulators. In addition, ALE analysis revealed additional cluster in the hypothalamus centered at MNI (2, −4, −12), with five studies contributed to this cluster (see Table 3, and Fig. 3B).

**Table 3:** Studies and relative foci coordinates in MNI space contributing to the identified clusters in employed meta-analyses on satiety regulators, using the activation likelihood estimation (ALE) method and Analysis of Brain Coordinates (ABC) approach.

ALE	ABC	Coordinates in MNI space		
*Contributors to Caudate cluster*		x	y	z
Page et al. (2009)	Page et al. (2009)	-10.5	8.2	12.7
Page et al. (2013)	Page et al. (2013)	-10.4	9.9	4.4
Lassman et al. (2010)	Lassman et al. (2010)	-14.1	18.4	5.1
Wolnerhassen et al. (2015)	Wolnerhassen et al. (2015)	-11.3	10.4	10.8
	Little et al. (2014)	-14.1	4.3	4.0
*Contributors to Hypothalamus cluster*				
Lassman et al. (2010)		-9.0	0.0	-7.7
Batterham et al. (2007)		-6.0	-11.94	-10.23
Page et al. (2009)		-3.0	-5.7	-9.8
Pannacciulli et al. (2007)		-4.0	-4.0	-19.32
Little et al. (2014)		-4.0	-1.0	-13

## 4 Discussion

This is the first systematic review and quantitative meta-analysis of functional neuroimaging data to determine which brain regions were most consistently activated in response to appetite/satiety regulators in healthy weight adults. We included studies that directly assessed brain activation in response to appetite/satiety regulators endogenously released, following acute food ingestion, and/or exogenously administered regulators. In addition, we employed two different coordinate-based meta-analysis approaches (ALE and ABC) to reveal convergent brain areas of neurohormonal gut-brain signaling to appetite/satiety regulators.

To our knowledge, there is only one previous systematic review that investigated the effects of appetite/satiety regulators on brain regions involved in appetite and satiety (Zanchi et al., 2017). However, that review (Zanchi et al., 2017) did not conduct functional neuroimaging meta-analysis to quantitively determine the concurrence/overlap of brain areas activated in response to appetite and satiety regulators across studies. In addition, data were combined from adult participants with a healthy weight and those living with obesity. Recent functional neuroimaging studies have shown that the brain activity in people with obesity is significantly different from that in subjects with a healthy weight in several brain regions implicated in food reward, with greater activation in the obese group paired with hypoactivity in areas associated with homeostatic satiety (Rothemund et al., 2007, Stoeckel et al., 2008, Szalay et al., 2012). Therefore, it is important to distinguish the role of appetite and satiety regulators in participants with a healthy weight from obese cohorts.

### 4.1 Concurrence of brain area modulated by appetite regulators

In support of our hypothesis, the systematic review revealed that the insula was one of the most reported regions across studies, with an overlap in 50% of the studies in response to appetite regulators. These results are in agreement with those of the previous systematic review (Zanchi et al., 2017). The insula is an important relay that connects the hypothalamus, OFC, and limbic system. It is often referred to as “ingestive cortex” because it contains primary taste neurons, projecting from the oral cavity (Scott and Plata-Salamán, 1999), as well as primary visceral afferents from the gut (Craig, 2002). The insula encodes multi-modal sensory features of foods (de Araujo et al., 2012), and its activity is modulated by hunger and satiety (de Araujo et al., 2006).

The systemic review also identified the amygdala, and OFC as key brain areas modulated with appetite regulators in 62% of the studies. The amygdala, and OFC encode motivation value of food cues (Jay et al., 2003). More specifically, the amygdala pass information about sensory cues onto the OFC and has an important role in reward processing (Padoa-Schioppa and Assad, 2008). Our results agree with the literature (Zanchi et al., 2017). However, due to the small number of studies identified for the coordinate based meta-analysis we could not conduct these analyses to assess the overlap quantitively in appetite regulators.

### 4.2 Concurrence of brain area modulated by satiety regulators

The hypothalamus is widely recognized as the gatekeeper to control food intake, highly influenced by nutrients, it is physically connected to other areas involved in maintaining homeostatic energy balance and receives projections from the gastrointestinal tract via the brainstem (Blouet and Schwartz, 2010). The ventromedial nucleus is the satiety center, and when stimulated, it causes the sensation of fullness, whereas the lateral hypothalamic area is the feeding center and when stimulated, it causes the sensation of hunger. The arcuate nucleus receives various signals from the gastrointestinal tract. This nucleus sends neuron fibers to regulate the feeding center and the satiety center. Previous neuroimaging studies have shown the hypothalamus is modulated by satiety and appetite (Gautier et al., 2001, Lizarbe et al., 2013, Smeets et al., 2005). Whilst CBF studies show a decrease in activation of the hypothalamus after the consumption of glucose (Page et al., 2013) and a high-fat meal (Eldeghaidy et al., 2016, Frank et al., 2012) compared with baseline, hunger showed to increase the hypothalamus activity (Lizarbe et al., 2013). Our systematic review and ALE meta-analysis revealed strong concurrence across studies and confirms the role of hypothalamus in appetite regulation. The results from the systematic review revealed negative correlation with satiety regulators, which is inconsistent with a previous systematic review that reported associations in opposite directions. These discrepancies might be due to variations in the inclusion criteria. Unlike Zanchi et al. (2017) we did not include participants with obesity or those below the age of 18 years. Functional neuroimaging studies showed greater activation in the group with obesity compared to healthy weight participants in areas associated with reward processing (Rothemund et al., 2007, Stoeckel et al., 2008, Szalay et al., 2012). In addition, the current focus was on the neuromodulation of appetite/satiety regulators in response to acute food intake, hence long-intervention studies were not included, unlike Zanchi et al. (2017)*,* which may cause discrepancies in findings.

The caudate nucleus is associated with perception of food stimuli, reward processing, and cognitive appetite control (Chen and Zeffiro, 2020). In this study, the systematic review identified a negative correlation between the activity of the caudate nucleus and satiety regulators, which was confirmed by the ALE and ABC meta-analysis methods. However, the results from the meta-analyses were combined across negatively and positively correlated studies. The relation of caudate nucleus to hunger and satiety is not yet clear, which perhaps explains the findings in this study. While some neuroimaging studies show reduction in caudate activity (Batterham et al., 2007, De Silva et al., 2011) in response to satiety regulators others show increased activity (Wolnerhanssen et al., 2015, Page et al., 2009). Increases in caudate activity after a meal could reflect top-down attentional control (Balleine et al., 2007), whereas the suppression after meal termination could be due to the dopamine-driven inhibition response (Mehta et al., 2012).

The thalamus is another key area revealed by this systematic review to modulate with satiety regulators. Thalamic brain activity has been reported to vary as a function of hunger or satiety (Tataranni et al., 1999), ghrelin application (Higgins et al., 2007) and glucose infusion (Jones et al., 2012, Little et al., 2014). The thalamus is the gateway to sensory perception, and it plays a major role in integrating proprioceptive information from the gastrointestinal tract (Kelley et al., 2005, Little et al., 2014) through the vagus nerve (Coss-Adame and Rao, 2014). The results from the systematic review demonstrate a correlation between thalamic activity with satiety regulators, which may reflect the role of food stimulation in modulating thalamus activity. This could be due to the role of the thalamus in integrating sensory perception (visual and taste cues) or the connection with the vagus nerve which sends information regarding the meal size and physical characteristics. The systematic review also revealed the association of insula with satiety regulators with 53% of the studies reported decreases in insula activation in response to satiety regulators.

### 4.3 Strengths and limitations of this review

To our knowledge this is the first study to employ functional neuroimaging meta-analysis to quantitatively define overlap of brain areas associated with satiety regulators across studies. The results from the generated activation maps of the meta-analysis (in our case from a total of 212 participants across the included studies) are more robust than those of any individual imaging study. The generated activation map from the healthy weight participants can be used as a reference or baseline to compare alterations with obesity, or in people with altered eating behavior. Another strength is the stringent and well-defined inclusion and exclusion criteria, which enabled an unbiased assessment of the central mechanisms regulating satiety and appetite. The present systematic review provides data and a clear overview of appetite neuroimaging findings in healthy weight participants.

One of the main limitations of this work is the relatively small number of appetite/satiety studies included in the meta-analysis, a consequence of the strict inclusion selection criteria. This did not allow us to perform sub-analyses on appetite/satiety regulators positively/negatively correlated with brain responses, or to investigate possible differences in neurohormonal gut-brain signaling in response to endogenously released appetite and satiety regulators compared with exogenously infused regulators. In addition, the small number of studies might be responsible for the absence of other key brain areas related to appetite and satiety regulations including the insula, thalamus and amygdala, OFC and ACC. The location of brain clusters/foci for the OFC and ACC activations varies widely across studies and this might also explain the lack of their concurrence in our analysis. Moreover, we stress that although a strict selection criterion was employed in this study, the presence of some unknown source of heterogeneity within the selected studies is possible, including individual differences in food preferences. We acknowledge that there are variations in the imaging modalities (task-based fMRI, resting-state fMRI and cerebral blood flow), study designs (endogenously released or exogenous administration), and food-related paradigms/stimulations (i.e., food images or taste stimuli) in the studies included, which might introduce some heterogeneity. Ideally, we would have separated the studies based on stimulation type and/or study design and/or imaging modality and performed separate analyses but this was not possible due to the small number of studies. Despite these variations and drawback, the robustness of our findings is supported by similar results obtained with two different approaches (ALE and ABC), particularly for the caudate convergence. In addition, variations/heterogeneity in the included study may lead to reduce the sensitivity of the meta-analysis, making it likely to be conservative, rather than causing false positive results/activations.

Finally, another limitation of this review was that the risk of bias analysis was performed using the Cochrane risk of bias guideline (Higgins et al., 2019). As this tool is not specifically designed for neuroimaging studies, the risk of bias might be underestimated (Acar et al., 2018).

## 5 Conclusions

In conclusion, our systematic review and quantitative meta-analyses add to the growing body of evidence describing brain areas involved in appetite and satiety processing. The generated brain activation maps for the gut-brain interactions in healthy weight participants can be used for comparison in future studies to define alterations with obesity and in pepole with altered eating behavior. We present robust evidence from the systematic review and two different coordinate based meta-analysis approaches/methods (ALE an ABC) for the importance of the hypothalamus and caudate nucleus in appetite and satiety processing. However, more work is needed to fully elucidate the complex interactions associated with the central regulation of appetite and satiety.

## Data Availability

Data associated with this paper will be available from SE upon request.
