# Supplementary material for: Mapping brain activity of gut-brain signaling to appetite and satiety in healthy adults: A systematic review and functional neuroimaging meta-analysis
Source: Neurosci Biobehav Rev. 2022 May;136:None. doi: 10.1016/j.neubiorev.2022.104603 (PMC9096878; doi:10.1016/j.neubiorev.2022.104603)
Supplement: Supplementary file 1 — Supplementary material [file mmc1.docx]

**Supplementary materials**

Supplementary Table 1. Search strategy.

| 1. "gut peptide" [MeSH terms]  2. "gut peptides" [MeSH terms]  3. "gut hormone" or "gastrointestinal hormone" [MeSH terms]  4. " gut hormones" or "gastrointestinal hormones" [MeSH terms]  5. "peptide YY " [MeSH terms]  6. "PYY" [MeSH terms]  7. "ghrelin" [MeSH terms]  8. "cholecystokinin" [MeSH terms]  9. "CCK" [MeSH terms]  10. "glucagon like peptide 1" [MeSH terms]  11. "GLP-1" [MeSH terms]  12. "satiety response" or "satiety" [MesSH terms]  13. "appetite" [MeSH terms]  14. "brain" [MeSH terms]  15. "imaging" [MeSH terms]  16. "neuroimaging" or "functional neuroimaging" or "neuroimaging" [MeSH terms]  17. "MRI" or "nuclear magnetic resonance imaging" [MeSH terms]  18. "fMRI" or "functional magnetic resonance imaging" [MeSH terms]  19. "PET" or "positron emission tomography" [MeSH terms]  20. "food" [MeSH terms]  21. "nutrient" [MeSH terms]  22. "nutrients" [MeSH terms]  23. "food intake" [MeSH terms]  24. "caloric intake" or "nutrient intake" [MeSH terms]  25. "meal" [MeSH terms]  26. "meals" [MeSH terms]  27. "meal intake" [MeSH terms]  28. "drink" [MeSH terms]  29. "drinks" [MeSH terms]  30. #1 or #2 or #3 or #4 or #5 or #6 or #7 or #8 or #9 or #10 or #11  31. #12 or #13  32. #14 or #15 or #16 or #17 or #18 or #19  33. #20 or #21 or #22 or #23 or #24 or #25 or #26 or #27 or #28 or #29  34. #30 and #31 and #32 and #33  35. #30 and #31 and #32 |
| --- |

**Supplementary Table 2.** Cochrane risk of bias assessment of the included studies.

| **Author., (year)** | **Appropriate cross-over design †** | **Randomized treatment order** | **Carry-over effect †** | **Unbiased data †** | **Allocation concealment** | **Blinding of participants and personnel** | **Blinding of outcome assessment** | **Incomplete outcome data** | **Selective outcome reporting** | **Other bias** |
| --- | --- | --- | --- | --- | --- | --- | --- | --- | --- | --- |
| Al-Zubaidi et al., (2019) * | Low | Unclear | Low | Low | Unclear | Unclear | Unclear | Unclear | Low | Low |
| **Batterham et al., (2007) *** | Low | Unclear | Low | Low | Unclear | Unclear | Unclear | Unclear | Low | Low |
| De Silva et al., (2011) * | Low | Unclear | Low | Low | Unclear | Unclear | Unclear | Unclear | Low | Low |
| **Dorton et al., (2017) *** | Low | Unclear | Low | Low | Unclear | Unclear | Unclear | Unclear | Low | Low |
| **Eldeghaidy et al., (2016) *** | Low | Unclear | Low | Low | Unclear | Unclear | Unclear | Low | Low | Low |
| **Gautier et al., (2000) **** | NA | Unclear | NA | NA | Unclear | Unclear | Unclear | Low | Low | Low |
| Heni et al., (2015) ** | NA | Unclear | NA | NA | Unclear | Unclear | Unclear | Unclear | Low | Low |
| Jakobsdottir et al., (2012) * | Low | Unclear | Low | Low | Unclear | Unclear | Unclear | Low | Low | Low |
| Jones et al., (2012) * | Unclear | Unclear | Unclear | Low | Unclear | Unclear | Unclear | Unclear | Low | Low |
| Kroemer et al., (2013) ** | NA | Unclear | NA | NA | Unclear | Unclear | Unclear | Unclear | Low | Low |
| Lassman et al., (2010) * | Unclear | Unclear | Unclear | Low | Unclear | Unclear | Unclear | Unclear | Low | Low |
| **Li et al., (2012) *** | Low | Unclear | Low | Low | Unclear | Unclear | Unclear | Unclear | Low | Low |
| **Little et al., (2014) *** | Unclear | Low | Unclear | Low | Unclear | Unclear | Unclear | Low | Low | Low |
| **Liu et al., (2000) *** | Low | Unclear | Unclear | Low | Unclear | Unclear | Unclear | Unclear | Low | Low |
| **Malik et al., (2008) **** | NA | Unclear | NA | NA | Unclear | Unclear | Unclear | Low | Low | Low |
| Page et al., (2009) * | Low | Unclear | Low | Low | Unclear | Unclear | Unclear | Unclear | Low | Low |
| Page et al., (2011) ** | NA | Unclear | NA | NA | Unclear | Unclear | Unclear | Unclear | Low | Low |
| **Page et al., (2013) *** | Low | Low | Low | Low | Unclear | Unclear | Unclear | Unclear | Low | Low |
| **Pannacciulli et al., (2007) **** | NA | Unclear | NA | NA | Unclear | Unclear | Unclear | Unclear | Low | Low |
| Schilling et al., (2014) * | Unclear | Unclear | Low | Low | Unclear | Unclear | Unclear | Unclear | Low | Low |
| **Spetter et al., (2014) *** | Low | Unclear | Low | Low | Unclear | Unclear | Unclear | Low | Low | Low |
| **Sun et al., (2014) *** | Low | Unclear | Low | Low | Unclear | Unclear | Unclear | Unclear | Low | Low |
| **Tataranni et al., (1999) **** | NA | Unclear | NA | NA | Unclear | Unclear | Unclear | Unclear | Low | Low |
| **Wolnerhanssen et al., (2015) *** | Low | Unclear | Low | Low | Unclear | Unclear | Unclear | Low | Low | Low |

†Domains specific only for cross-over study design. *Cross-over trials. **Controlled trials. NA; not applicable.

Supplementary Table 3. List of reported brain areas in the systematic review. This list included brain areas correlated with endogenously released or exogenously administered appetite or satiety regulators.

| **Brain areas** | **Number of studies (percentage)** | **Reference** |
| --- | --- | --- |
| ***Brain areas correlated positively with appetite regulators (8 studies)*** | | |
| Amygdala | 5 (62.5%) | **Malik et al., (2008); Jones et al., (2012); Kroemer et al., (2013); Li et al., (2012); Sun et al., (2014)** |
| OFC | 5 (62.5%) | **Malik et al., (2008); De Silva et al., (2011); Li et al. (23); Goldstone et al., (2014); Sun et al., (2014)** |
| Insula | 4 (50%) | **Malik et al., (2008); Jones et al. (2012); Li et al., (2012); Sun et al., (2014)** |
| Hippocampus | 4 (50%) | **Malik et al., (2008); De Silva et al., (2011); Jones et al., (2012); Goldstone et al., (2014)** |
| Palldium | 3 (37.5%) | **Kroemer et al., (2013);** Li et al., (2012); Sun et al., (2014) |
| Midbrain | 3 (37.5%) | Jones et al., (2012); **Kroemer et al., (2013);** Sun et al., (2014) |
| Hypothalamus | 3 (37.5%) | Batterham et al., (2007); Jones et al. (2012); **Kroemer et al., (2013)** |
| Fusiform gyrus | 2 (25%) | Malik et al., (2008); **Kroemer et al., (2013)** |
| Thalamus | 2 (25%) | Jones et al., (2012); **Kroemer et al., (2013)** |
| Caudate | 2 (25%) | Malik et al., (2008); **Kroemer et al., (2013)** |
| Brainstem | 2 (25%) | Batterham et al. (2007); Jones et al. (2012) |
| VTA | 2 (25%) | Malik et al., (2008); Batterham et al., (2007) |
| ***Brain areas correlated positively with satiety regulators (8 studies)*** | | |
| ACC | 3 *(*37.5%) | **Batterham et al., (2007); Page et al., (2009); Page et al., (2011)** |
| Putamen | 3 (37.5%) | **Batterham et al., (14); Page et al., (26); Schilling et al., (29)** |
| Insula | 2 (25%) | Batterham et al., (2007); Schilling et al., (2014) |
| Caudate | 2 (25%) | Page et al., (2009); Schilling et al., (2014) |
| Hypothalamus | 2 (25%) | Batterham et al., (2007); Page et al., (2011) |
| Inferior frontal gyrus | 2 (25%) | Page et al., (2009); Schilling et al. (2014) |
| Superior temporal gyrus | 2 (25%) | Batterham et al., (2007); Page et al., (2009) |
| ***Brain areas negatively correlated with satiety regulators (15 studies)*** | | |
| Insula | 8 (**53%)** | Al-Zubaidi et al., (2019)**;** De Silva et al., (2011)**;** Eldeghaidy et al., (2016)**;** Jakobsdottir et al., (2012)**; Li et al., (2012);** Schilling et al., (2014); Spetter et al., (2014)**;** Sun et al., (2014) |
| Hypothalamus | 5 (33%) | Lassman et al., (2010); Liu et al. (2000); Page et al., (2009); Spetter et al. (2014)**;** Page et al. (2013) |
| OFC | 4 (26**%)** | De Silva et al., (2011); Heni et al., (2015); **Li et al., (2000)***;* Gautier et al., (2000) |
| Thalamus | 4 (**26%)** | Eldeghaidy et al., (2016); Lassman et al., (2010); **Li et al., (2012***);* Gautier et al., (2000) |
| Putamen | 4 (**26%)** | Spetter et al., (2014)**;** Page et al., (2013)**;** Sun et al., (2014)**;** Gautier et al., (2000) |
| Caudate | 3 (**20%)** | Lassman et al., (2010); **Li et al., (2000);** Page et al., (2013) |
| Cerebellum | 3 (**20%)** | Eldeghaidy et al., (2016); Lassman et al., (2010); Page et al., (2009) |
| Temporal gyrus | 2 (13%) | Eldeghaidy et al., (2016); Lassman et al., (2010) |

ACC, anterior cingulate cortex; OFC, orbitofrontal cortex; VTA, ventral tegmental area.
